# Substantia nigra-related gene polymorphisms associated with acute antipsychotic-induced movement disorders

**DOI:** 10.1186/s40779-025-00652-w

**Published:** 2025-10-02

**Authors:** Kenji Hashimoto

**Affiliations:** 1 Chiba University Center for Forensic Mental Health, Chiba, 260-8670 Japan; 2 Department of Anesthesiology, Pain and Perioperative Medicine, the First Affiliated Hospital of Zhengzhou University, Zhengzhou, 450052 China; 3 Basic Medicine Research Innovation Center for Cardiometabolic Diseases, Ministry of Education, Southwest Medical University, Luzhou, 646000 Sichuan China

**Keywords:** Antipsychotic, Movement disorder, Polymorphism, Substantia nigra

Antipsychotics, especially many second-generation antipsychotics (SGAs), remain central to schizophrenia treatment, are indispensable in acute mania and for bipolar maintenance (with selected roles in bipolar depression), and serve as evidence-based augmenters in treatment-resistant depression. Nonetheless, acute antipsychotic-induced movement disorders (AIMDs) [extrapyramidal symptoms (EPS)] are common and clinically costly, impairing quality of life, adherence, and outcomes. The acute spectrum is dominated by dystonia (sustained, often painful contractions), akathisia (subjective restlessness with motor drive), and drug-induced Parkinsonism (bradykinesia, rigidity, tremor), typically arising within hours to weeks of initiation or dose increase, distinct from tardive syndromes that emerge after months to years [1]. Across observational cohorts, roughly one-third of treated patients experience AIMDs (approximately 37%), highlighting the need for prevention and early detection [2]. Risk is higher with strong D2 receptor blockade and higher exposure (notably high-potency first-generation drugs, but also some SGAs at greater doses), rapid titration, younger age for dystonia, and female/older age for Parkinsonism. Although SGAs reduce risk on average, network analyses still show increased akathisia or greater use of antiparkinsonian drugs versus placebo for many agents [2, 3]. Mechanistically, higher nigrostriatal D2 occupancy is the proximal driver; positron emission tomography (PET) studies show that therapeutic striatal D2 occupancy is 65 − 80% [2, 3]. Exceeding 80% markedly increases AIMD risk [3]. Yet occupancy alone does not explain phenotype diversity or inter-individual liability, and the genetic basis has been unclear.

Lu et al. [4] report the large-scale genome-wide association study (GWAS) focused on early AIMDs, leveraging a Han Chinese discovery set (*n* = 2016), an independent paliperidone monotherapy cohort (*n* = 277), and multi-ancestry validation in the clinical antipsychotic trials of intervention effectiveness (CATIE) (*n* = 766). Over 6 weeks, Parkinsonism (Simpson-Angus), akathisia (Barnes), and involuntary movements [Abnormal Involuntary Movement Scale (AIMS)] were prospectively assessed. The study identified genome-wide signals in *RAB44* (rs116249243, rs117097482) for EPSs, a lead variant for akathisia (rs6826172), and numerous loci for involuntary movements (mapping to 11 genes). Associations in *CNTNAP2, LUZP2, TMEM167A*, and *RAB44* replicated in the paliperidone sample; *RAB44* also replicated in CATIE. Gene-based tests highlighted *XRCC4* and *PAIP2B* (involuntary movements), and polygenic risk score (PRS) models showed modest, preliminary discrimination. Post-GWAS enrichment pointed to substantia nigra-enriched signals, transcriptome-wide association study (TWAS) linked *XRCC4* expression in basal ganglia, converging on nigrostriatal vulnerability without implying causality. These findings refine the classic model: D2 blockade sets the stage, but genetically mediated differences in dopaminergic neuron function, synaptic organization, or neurodevelopment/DNA-repair pathways (e.g., *XRCC4*) may determine whether a given occupancy produces circuit-level dysfunction and clinical EPSs [4]. In this study, the robust *RAB44* signal motivates testing cellular-trafficking hypotheses (and potential peripheral contributions) in AIMD susceptibility. Signals in connectivity-linked genes (*CNTNAP2*, *LUZP2*) and overlaps with Parkinson’s-related biology further suggest shared vulnerability pathways. Phenotype specificity is notable: the akathisia lead (rs6826172) showed its strongest effects on akathisia items in both discovery and CATIE; although intergenic, it is an expression quantitative trait locis (eQTL) for *GLRB*, implicating glycinergic inhibitory tone in akathisia risk.

Clinically, PRS performance is encouraging but not yet practice-ready. AIMD liability is multifactorial, integrating drug/dose, titration rate, co-medications (e.g., anticholinergics, β-blockers), iron status, and demographics alongside genetics. Priority next steps are prospective studies in first-episode, antipsychotic-naive patients that combine pharmacokinetics, positron emission tomography-based occupancy, and multi-omics with granular, syndrome-specific phenotyping. In the interim, prevention and early response remain paramount: use conservative initial dosing and gradual titration, select lower-EPSs options when possible, avoid rapid escalation/high occupancy, and apply standardized scales during the first weeks. Acute dystonia should be treated immediately with parenteral anticholinergics, followed by a brief oral course. Drug-induced Parkinsonism is managed by reducing the dose or switching antipsychotics; a short course of anticholinergic or amantadine may be added if needed. Akathisia is addressed with dose adjustment plus evidence-supported adjuncts (e.g., propranolol), while avoiding misattribution to agitation or anxiety [5].

This multi-cohort GWAS of AIMDs identifies novel risk loci with convergent gene/TWAS signals and substantia nigra enrichment, and shows preliminary PRS utility for early risk stratification. Key limitations include restricted drug coverage (replication limited to paliperidone), predominance of antipsychotic-exposed patients with concomitant antiparkinsonian use, exclusion of baseline AIMDs, limited trajectory capture, and a modest total sample for genome-scale discovery. Future work should span first-episode/naïve cohorts, multiple antipsychotics, longitudinal phenotyping, functional validation of implicated genes, and trials of genotype-guided prevention or prescribing.

In conclusion, this multi-cohort, multi-ancestry study links substantia nigra-related polymorphisms (including *RAB44* and others) to early AIMDs, Parkinsonism, dystonia, and akathisia, shifting the field from occupancy thresholds toward tractable biology. The work nominates specific genes and pathways for functional follow-up and lays the groundwork for future risk-stratified prescribing. Next steps include prospective replication across antipsychotic classes and dosing ranges, and development of ancestry-aware polygenic clinical risk scores evaluated in pragmatic trials of genotype-guided dosing. Mechanistic studies in iPSC-derived dopaminergic neurons and microglia, coupled with CRISPR perturbation and single-cell omics, will be critical to establishing causality and prioritizing therapeutic targets. Until predictive tools mature, careful dosing, early monitoring, correction of modifiable risks (e.g., iron deficiency, interactions), and timely, phenotype-specific treatment remain the most effective strategies to lessen the burden of AIMDs.

## Data Availability

Not applicable.

## Abbreviations

AIMDs: Antipsychotic-induced movement disorders
CATIE: Clinical antipsychotic trials of intervention effectiveness
EPS: Extrapyramidal symptoms
GWAS: Genome-wide association study
PRS: Polygenic risk scores
SGA: Second-generation antipsychotics
